# Supplementary material for: Modeling glioblastoma heterogeneity as a dynamic network of cell states
Source: Mol Syst Biol. 2021 Sep 16;17(9):e10105. doi: 10.15252/msb.202010105 (PMC8444284; doi:10.15252/msb.202010105)
Supplement: Supplementary file 6 — Source Data for Figure 5 [file MSB-17-e10105-s004.zip › Figure5A_sourcedata/GSEA_3071/hallmarks_stateI.GseaPreranked.1623742531165/HALLMARK_INFLAMMATORY_RESPONSE.html]

Details for gene set HALLMARK\_INFLAMMATORY\_RESPONSE[GSEA]

|  || Dataset | state13071 |
| Phenotype | NoPhenotypeAvailable |
| Upregulated in class | na\_pos |
| GeneSet | HALLMARK\_INFLAMMATORY\_RESPONSE |
| Enrichment Score (ES) | 0.31193823 |
| Normalized Enrichment Score (NES) | 1.5956354 |
| Nominal p-value | 0.02793296 |
| FDR q-value | 0.039159838 |
| FWER p-Value | 0.347 |
Table: GSEA Results Summary

  

Fig 1: Enrichment plot: HALLMARK\_INFLAMMATORY\_RESPONSE      
 Profile of the Running ES Score & Positions of GeneSet Members on the Rank Ordered List

  

| PROBE | GENE SYMBOL | GENE\_TITLE | RANK IN GENE LIST | RANK METRIC SCORE | RUNNING ES | CORE ENRICHMENT || 1 | MMP14 |  |  | 27 | 1.526 | 0.0405 | Yes |
| 2 | TNFAIP6 |  |  | 36 | 1.470 | 0.0847 | Yes |
| 3 | TIMP1 |  |  | 47 | 1.381 | 0.1254 | Yes |
| 4 | TPBG |  |  | 51 | 1.346 | 0.1671 | Yes |
| 5 | NMI |  |  | 131 | 0.962 | 0.1747 | Yes |
| 6 | INHBA |  |  | 140 | 0.941 | 0.2022 | Yes |
| 7 | EMP3 |  |  | 260 | 0.726 | 0.1907 | Yes |
| 8 | ATP2B1 |  |  | 270 | 0.713 | 0.2107 | Yes |
| 9 | IL18 |  |  | 272 | 0.710 | 0.2329 | Yes |
| 10 | IRF1 |  |  | 276 | 0.705 | 0.2543 | Yes |
| 11 | TAPBP |  |  | 287 | 0.692 | 0.2733 | Yes |
| 12 | ABCA1 |  |  | 345 | 0.633 | 0.2768 | Yes |
| 13 | TLR3 |  |  | 359 | 0.625 | 0.2928 | Yes |
| 14 | TNFSF10 |  |  | 395 | 0.590 | 0.3014 | Yes |
| 15 | NAMPT |  |  | 456 | 0.544 | 0.3013 | Yes |
| 16 | PCDH7 |  |  | 478 | 0.529 | 0.3119 | Yes |
| 17 | CD70 |  |  | 610 | 0.446 | 0.2882 | No |
| 18 | IL15 |  |  | 635 | 0.439 | 0.2951 | No |
| 19 | KLF6 |  |  | 647 | 0.433 | 0.3056 | No |
| 20 | BST2 |  |  | 677 | 0.421 | 0.3106 | No |
| 21 | CD55 |  |  | 726 | 0.403 | 0.3095 | No |
| 22 | CX3CL1 |  |  | 828 | 0.373 | 0.2920 | No |
| 23 | LPAR1 |  |  | 829 | 0.372 | 0.3038 | No |
| 24 | ITGA5 |  |  | 874 | 0.357 | 0.3024 | No |
| 25 | ACVR2A |  |  | 934 | 0.343 | 0.2962 | No |
| 26 | MET |  |  | 974 | 0.333 | 0.2955 | No |
| 27 | C3AR1 |  |  | 984 | 0.330 | 0.3033 | No |
| 28 | LY6E |  |  | 1030 | 0.321 | 0.3005 | No |
| 29 | ITGB3 |  |  | 1102 | 0.306 | 0.2896 | No |
| 30 | SGMS2 |  |  | 1135 | 0.301 | 0.2899 | No |
| 31 | MXD1 |  |  | 1151 | 0.298 | 0.2950 | No |
| 32 | ABI1 |  |  | 1200 | 0.288 | 0.2902 | No |
| 33 | IFNAR1 |  |  | 1214 | 0.286 | 0.2955 | No |
| 34 | SELENOS |  |  | 1287 | 0.270 | 0.2832 | No |
| 35 | SLC7A1 |  |  | 1389 | 0.254 | 0.2621 | No |
| 36 | HRH1 |  |  | 1541 | -0.261 | 0.2267 | No |
| 37 | CDKN1A |  |  | 2083 | -0.332 | 0.0808 | No |
| 38 | PDE4B |  |  | 2132 | -0.339 | 0.0777 | No |
| 39 | RAF1 |  |  | 2213 | -0.356 | 0.0658 | No |
| 40 | CALCRL |  |  | 2249 | -0.363 | 0.0672 | No |
| 41 | BTG2 |  |  | 2318 | -0.375 | 0.0594 | No |
| 42 | DCBLD2 |  |  | 2407 | -0.393 | 0.0464 | No |
| 43 | KCNJ2 |  |  | 2528 | -0.422 | 0.0251 | No |
| 44 | IL7R |  |  | 2665 | -0.460 | 0.0003 | No |
| 45 | ITGB8 |  |  | 2840 | -0.516 | -0.0336 | No |
| 46 | SERPINE1 |  |  | 3050 | -0.634 | -0.0740 | No |
| 47 | EDN1 |  |  | 3079 | -0.660 | -0.0612 | No |
| 48 | RGS16 |  |  | 3134 | -0.708 | -0.0544 | No |
| 49 | SRI |  |  | 3177 | -0.757 | -0.0426 | No |
| 50 | CD82 |  |  | 3261 | -0.870 | -0.0391 | No |
| 51 | SLC1A2 |  |  | 3463 | -1.714 | -0.0430 | No |
| 52 | PDPN |  |  | 3472 | -1.788 | 0.0113 | No |
Table: GSEA details [plain text format]

  

Fig 2: HALLMARK\_INFLAMMATORY\_RESPONSE: Random ES distribution      
 Gene set null distribution of ES for **HALLMARK\_INFLAMMATORY\_RESPONSE**
